# High-Throughput Cultivation for the Selective Isolation of Acidobacteria From Termite Nests

**DOI:** 10.3389/fmicb.2020.597628

**Published:** 2020-11-06

**Authors:** Markus Oberpaul, Celine M. Zumkeller, Tanja Culver, Marius Spohn, Sanja Mihajlovic, Benedikt Leis, Stefanie P. Glaeser, Rudy Plarre, Dino P. McMahon, Peter Hammann, Till F. Schäberle, Jens Glaeser, Andreas Vilcinskas

**Affiliations:** ^1^Branch for Bioresources, Fraunhofer Institute for Molecular Biology and Applied Ecology (IME), Giessen, Germany; ^2^Institute of Applied Microbiology, Justus Liebig University Giessen, Giessen, Germany; ^3^Bundesanstalt für Materialforschung und -prüfung, Berlin, Germany; ^4^Institute of Biology, Free University of Berlin, Berlin, Germany; ^5^Sanofi-Aventis Deutschland GmbH, R&D Integrated Drug Discovery, Hoechst Industrial Park, Frankfurt am Main, Germany; ^6^Institute for Insect Biotechnology, Justus Liebig University Giessen, Giessen, Germany

**Keywords:** termites, *Coptotermes*, core microbiome, natural products discovery, Acidobacteria, underexplored phyla, social insects, termite-associated microbes

## Abstract

Microbial communities in the immediate environment of socialized invertebrates can help to suppress pathogens, in part by synthesizing bioactive natural products. Here we characterized the core microbiomes of three termite species (genus *Coptotermes*) and their nest material to gain more insight into the diversity of termite-associated bacteria. Sampling a healthy termite colony over time implicated a consolidated and highly stable microbiome, pointing toward the fact that beneficial bacterial phyla play a major role in termite fitness. In contrast, there was a significant shift in the composition of the core microbiome in one nest during a fungal infection, affecting the abundance of well-characterized *Streptomyces* species (phylum Actinobacteria) as well as less-studied bacterial phyla such as Acidobacteria. High-throughput cultivation in microplates was implemented to isolate and identify these less-studied bacterial phylogenetic group. Amplicon sequencing confirmed that our method maintained the bacterial diversity of the environmental samples, enabling the isolation of novel Acidobacteriaceae and expanding the list of cultivated species to include two strains that may define new species within the genera *Terracidiphilus* and *Acidobacterium*.

## Introduction

Subterranean termites play a key role in the decomposition of plant biomass (Scheffrahn et al., 2015; Kuwahara et al., 2017). Their ingestion and degradation of wood can change the composition of soils and remodel entire landscapes (Bonachela et al., 2015). Furthermore, some termites cause damage valued at more than US$ 22 billion p.a. by attacking wooden structures (Chouvenc et al., 2011; Chouvenc et al., 2016). The genus *Coptotermes* contains the largest number of economically destructive termite species (Scheffrahn et al., 2015), including *C. formosanus* (Su, 2003) and *C. gestroi* (Jenkins et al., 2007; Vargo and Husseneder, 2009), both of which are native to Asia but have spread to other areas as invasive pests. Furthermore, *C. testaceus* is the dominant termite species infesting living trees in the central Amazonian rain forests (Apolinário and Martius, 2004).

Termites are eusocial insects with worker, soldier and reproductive castes. Their social lifestyle includes allogrooming and trophallaxis, which requires frequent direct contact among individuals and facilitates the spread of microbes (He et al., 2018). Accordingly, termites have evolved behaviors to prevent infections, such as the removal of corpses from the nest (Davis et al., 2018). Furthermore, more than 150 million years of coevolution has established a beneficial relationship between termites and their surrounding microbial community (Bourguignon et al., 2015; Legendre et al., 2015). These microbes not only facilitate the digestion of wood (Long et al., 2010; Poulsen et al., 2014; Wang et al., 2016), but also suppress the growth of entomopathogenic bacteria and fungi (Traniello et al., 2002; Chouvenc et al., 2013; Mevers et al., 2017).

Social insects—especially termites—are protected by parts of the symbiotic and stable microbiota, particularly natural products (NPs) producing organisms, therefore they are discussed as a fruitful source for NPs discovery (Klassen et al., 2019). Such specialized microbial communities are essential for the lifestyle of social insects (Audisio et al., 2005; Kaltenpoth, 2009; Chouvenc et al., 2018). For example, several actinomycete mutualists associated with leaf-cutting ants produce antifungal natural products such as dentigerumycin or antimycotic polyenes (Haeder et al., 2009; Devine et al., 2017). Similar mechanisms have been reported for higher and lower termites (Stroeymeyt et al., 2014; Benndorf et al., 2018). Termite soldiers not only prevent invasion by predators (Husseneder and Simms, 2014), but they also possess a broad range of chemical defense mechanisms including the production of antibacterial agents (Sobotnik et al., 2010; Terrapon et al., 2014; He et al., 2018). Bacteria that synthesize antimicrobial compounds have been isolated from the nest material of *C. formosanus* (Mevers et al., 2017; Chouvenc et al., 2018). Bacterial genera known to form symbiotic interactions with social insects include *Streptomyces* and *Pseudonocardia*, both of which are actinomycetes known to synthesize natural products (Chouvenc et al., 2013). However, termites are also associated with *Burkholderia* species, representing the well-characterized phylum Proteobacteria (Santos et al., 2004), as well as less-studied phyla with the potential to synthesize as yet unexplored natural products (Makonde et al., 2015; Su et al., 2016). A balanced consortium of beneficial microbes in the environment is therefore necessary to maintain the health of the colony (Chouvenc et al., 2011; Rosengaus et al., 2011; Peterson and Scharf, 2016).

Here we carried out a systematic analysis of the microbial core community at different levels in the nest of three *Coptotermes* species, revealing the stability of the core microbiome and its impact on colony fitness. Selected bacterial strains were enriched and cultivated in a high-throughput microplate-based format to gain insight into the roles of less-studied bacterial phyla that are generally underrepresented in culture, such as the Acidobacteria.

## Materials and Methods

### Termite Ancestry, Rearing, and Sampling

Captive colonies of *C. testaceus* (± facing fungal burden), *C. formosanus* and *C. gestroi* were reared for more than 15 years at the Federal Institute for Materials Research and Testing in Berlin (BAM) in separate metal tanks with a volume of ∼2 m^3^ (Supplementary Figure 1). *C. gestroi* was reared at 26 ± 2°C, 87 ± 5% relative humidity (*RH*), and was fed on birch wood, which was refreshed every 3 months. *C. testaceus* and *C. formosanus* were reared at 29 ± 2°C, 75 ± 5% *RH*, and were fed on pine wood as above. All three species were identified based on their morphological characters (Scheffrahn et al., 2015).

Samples from the carton nest material of *C. testaceus* were collected at four different time points over 2 years. Sterile plastic spatulas were used to transfer 200–300 g of nest material into sterilized glass Petri dishes. At first, surface material was collected (*surface*), then wood pieces from feeding events from below the nest surface were placed aside and biofilms were sampled from the fresh eroded base of the wood pieces (*wood*) (Supplementary Figure 2). Autoclaved steel double spatulas were used to excavate sample material from the carton nest (*carton nest*). For all termite samples, first, filter carton paper traps were placed in the nests to separate the termite soldiers from the nest material. Using soft tweezers, the termite specimens were collected in 50 mL tubes and directly frozen (*termites*). All samples were frozen and stored at –50°C for processing and at 4°C for cultivation purposes.

### Sample Processing and Environmental DNA Extraction From Nest Material and Termites

Nucleic acids were extracted using the NucleoSpin soil DNA purification kit (Macherey Nagel, Düren, Germany) to combine a gentle mechanical and chemical disruption of samples. To increase the yield, 200–500 mg of nest material or 12–28 termites as a whole were weighed into the NucleoSpin bead tubes before adding 700 μL of lysis buffer SL2. The tubes were vortexed horizontally for 15 min at 40 Hz using a Top Mix 11118 (Fisher Scientific, Schwerte, Germany) and then centrifuged at 12,000 × *g* for 2 min. Thereby, the samples were mechanically broken and lysed. Subsequent extraction steps were carried out according to the manufacturer’s recommendations. Finally, the yield and purity of the received DNA from the termites and from nest material was checked using a NanoDrop ND-1000 UV/Vis spectrophotometer (Thermo Fisher Scientific, Waltham, MA, United States). In the following, we will use the term environmental DNA (eDNA) for these DNA samples and differentiate between the origin (e.g., termites and nest material).

### 16S rRNA Gene Amplicon Sequencing and Data Processing

PCR amplification and Illumina 300 bp paired-end read sequencing of the eluted eDNA extracts were carried out by LGC Genomics (Berlin, Germany) using an Illumina (San Diego, CA, United States) MiSeq V3 system. The variable V3-V4 region was amplified using forward primer U341F (5′-CCT AYG GGR BGC ASC AG-3′) and reverse primer U806R (5′-GGA CTA CNN GGG TAT CTA AT-3′) (Klindworth et al., 2013). Data pre-processing, including the demultiplexing of all libraries, was carried out using Illumina bcl2fastq v1.8.4. Reads were sorted by amplicon inline barcodes unique to each sample, allowing one mismatch per barcode and discarding those with missing or one-sided barcodes or conflicting barcode pairs. Reads with a final length < 100 bases were discarded during the clipping of the sequencing adapter from all reads. During primer detection and clipping, three mismatches were allowed per primer, and pairs of primers had to be present in each sequence fragment. If primer-dimers were detected, the outer primer copies were clipped from the sequence. The sequence fragments were converted to forward-reverse primer orientations after removing the primer sequences. The forward and reverse reads were combined using BBMerge v34.48^1^.

Each read was aligned using the SILVA Incremental Aligner (SINA v1.2.10 for ARB SVN revision 21008) (Pruesse et al., 2012) against the SILVA SSU rRNA SEED and quality controlled (Quast et al., 2013). Reads < 300 aligned nucleotides and reads with >2% of ambiguities, or 2% of homopolymers, respectively, were excluded from further analysis. All reads containing a low alignment quality (50 alignment identity, 40 alignment score reported by SINA), were identified and excluded from downstream processing. After this quality control, identical reads were dereplicated, the unique reads were clustered (OTUs), on a per sample basis, and the reference read of each OTU was classified. Dereplication and clustering was done using cd-hit-est (version 3.1.2)^2^ (Li and Godzik, 2006) running in accurate mode, ignoring overhangs, and applying identity criteria of 1.00 and 0.98, respectively.

Classification was achieved by running a local nucleotide BLAST search against the non-redundant version of the SILVA SSU Ref dataset release 132^3^ using blastn v2.2.30+^4^ with standard settings (Camacho et al., 2009).

The classification of each OTU reference read was mapped onto all reads that were assigned to the respective OTU. This yields quantative information (number of individual reads per taxonomic path), within the limitations of PCR and sequencing technique biases, as well as, multiple rRNA operons. Reads without any BLAST hits or reads with weak BLAST hits, where the function “(% sequence identity + % alignment coverage)/2” did not exceed the value of 93, remained unclassified. These reads were assigned to the meta group “No Relative” in the SILVAngs fingerprint and Krona Charts (Ondov et al., 2011)^5^. Data were corrected by excluding all reads affiliated to Archaea (5.7%), chloroplasts (0.003%), mitochondria (0.003%), Eukaryota (3.4%), or *No Relative* (0.25%) from further analysis (9.35% excluded in total). Reads affiliated to the domain Bacteria were set to 100%.

### Statistical Analysis

Statistical evaluation was carried out using PAST v3.18^6^ (Hammer et al., 2001) including non-metric multidimensional scaling (nMDS) (Taguchi and Oono, 2005) and one–way analysis of similarities (ANOSIM) via 9999 permutations with a statistical significance test (Clarke, 1993) at genus-level resolution for operational taxonomic units (OTUs) assigned to phylogenetic groups, representing clusters of uncultivated bacteria or genera. ANOSIM and nMDS scores were computed using the Bray-Curtis similarity index. The calculations were used to illustrate the ratio between within-group and between-group dissimilarities of microbial communities associated with nest materials, termite samples, different sampling time points, before and after a 5 μm filtration step during Nycodenz density gradient centrifugation. A ternary plot and heat map charting the relative abundance of phylogenetic groups were used to find overlapping bacterial genera among the three *Coptotermes* species and to address which nest level represents the best source of underexplored bacterial phyla. We calculated diversity indices such as Chao 1, Shannon, dominance, and evenness, considering the number of phylogenetic groups and the number of individual reads per phylogenetic group to show similarities within the non-infected nest material and differences among all samples (Harper, 1999).

### Retrieval of Living Cells From Nest Material Using Nycodenz Density Centrifugation

Living cells were retrieved from the *C. testaceus* carton nest material by density gradient centrifugation using a 60% (w/v) Nycodenz solution (Axis Shield, Dundee, United Kingdom) as previously described (Hevia et al., 2015). We transferred 1.0–2.0 g of nest material aseptically into four 50-mL tubes, and added 20 mL of autoclaved Milli-Q ultrapure water to each tube. The samples were homogenized three times at 225 Hz for 5 s using an S25 KD 18 G dispersal tool connected to an Ultra-Thurrax T25 basic (both provided by IKA Werke, Staufen im Breisgau, Germany) to a fineness of 10–50 μm. We added another 10 mL of Milli-Q ultrapure water to each tube and centrifuged briefly at 450 × *g* at 4°C using an A4-81 swing-out rotor (Eppendorf, Hamburg, Germany). We then transferred 25 mL of the debris-free supernatant to a fresh 50-mL tube. The homogenate was carefully underlain with the autoclaved 60% Nycodenz solution. The tubes were then centrifuged at 3,050 × *g* for 60 min at 4°C in a swing-out rotor without acceleration and deceleration to form the desired layer of bacteria (Berry et al., 2003). The layer containing the bacteria was passed through a 5-μm cellulose acetate Minisart syringe filter (Sartorius, Göttingen, Germany) and collected in a 5-mL reaction tube.

### Cultivation Media

For the cultivation of Acidobacteria from the *C. testaceus* carton nest material, we used VL55 medium (de Castro et al., 2013) (DSMZ no. 1266) supplemented with 0.05% (w/v) xylan instead of glucose (Sait et al., 2002) and used FeCl_3_⋅6 H_2_O instead of FeCl_2_⋅4 H_2_O for trace element solution SL-10 (Tschech and Pfennig, 1984).

### Microplate-Based Cultivation

To estimate the total number of cells per well, the Nycodenz cell phase was diluted 1,000-fold in phosphate buffered saline (PBS) and analyzed by flow cytometry on a FACSCalibur (BD Bioscience, San Jose, CA, United States) following the protocol of the Bacteria Counting Kit (B7277, Thermo Fisher Scientific) and visualized using FlowJo v10.4.2 (FlowJo, Ashland, OR, United States). Based on a combination of cell enumeration and the live/dead ratio, calculated using the LIVE/DEAD BacLight Bacterial Viability Kit (L34856, Thermo Fisher Scientific), 40 μL of cell suspension containing an average of 40 cells was distributed using a Matrix WellMate (Thermo Fisher Scientific) into 64 × 384-well microplates (Greiner Bio-One, Kremsmünster, Austria) minus the media controls on each plate (24,384 wells in total). Following incubation for up to 14 days at 28°C, 70 ± 5% *RH*, growth was verified by measuring the optical density at 600 nm (OD_600_) in a Wallac 1420 Victor2 Microplate Reader (Perkin Elmer, Waltham, MA, United States). The threshold was set by averaging the media controls on each plate to establish the media background. All cultures with verified growth were automatically transferred into 96-deepwell microplates (Corning, New York, NY, United States) pre-filled with 1.5 mL medium using a Precision XS liquid-handling system (BioTek Instruments, Bad Friedrichshall, Germany). Then, plates were incubated at 28°C using a Duetz System holder (Adolf Kühner, Birsfelden, Switzerland), shaking at 220 rpm with 2.5 cm deflection for 7 days.

### Rapid Identification of Cultures by 16S rRNA Gene Sequencing

Culture broth from the above mentioned cultivation step was divided into aliquots for OD_600_ measurements, DNA extraction, and cryo-conservation using the VIAFLO 384 system (Integra Biosciences, Zizers, Switzerland). Glycerol stocks were prepared by first pre-filling tubes with 300 μL 80% glycerol using the Matrix Wellmate and then adding 200 μL of culture broth. For DNA extraction, 200 μL of broth was transferred to microtubes (Qiagen, Hilden, Germany) containing 2.3-mm zirconia beads (Carl Roth, Karlsruhe, Germany) and the cells were disrupted by 2 × 1 min pulses at 30 Hz using a TissueLyser II (Qiagen). The tubes were centrifuged for 2 min at 4,000 × *g*, incubated at 70°C for 45 min and centrifuged again as above. The supernatant was used for 16*S* rRNA gene amplification with primer pair E8F (5′-GAG TTT GAT CCT GGC TCA G-3′) and 1492R (5′-ACG GYT ACC TTG TTA CGA CTT-3′) (Lane, 1991).

### Phylogenetic Classification of Isolated Acidobacteria

Liquid cultures affiliated to Acidobacteria via 16*S* rRNA sequencing (only using the reverse primer), were prioritized to obtain pure cultures. Thus, the liquid cultures were streaked onto solid VL55 medium containing 1.5% (w/v) agar no. 1 (Oxoid Deutschland, Wesel, Germany) and in parallel onto Reasoner’s 2A medium (R2A) prepared from DSMZ no. 830 by reducing the pH to 5.5 using 1.95 g/L 2-(*N*-morpholino)ethanesulfonic acid (MES) (Sigma-Aldrich, St. Louis, MO, United States). The 16*S* rRNA gene sequence of FhG110202 was used to detect the 40 most similar sequences from the NCBI 16*S* ribosomal RNA sequences database using BLAST^7^. Multiple sequences were aligned using ClustalW with default parameters, including nearly full-length 16*S* rRNA gene sequences of FhG110206, FhG110214 and representatives of different subgroups of Acidobacteria. The phylogenetic tree was calculated using MEGA v7.0.26^8^, by applying the maximum-likelihood method using the Tamura-Nei model (Kumar et al., 2016) with 1,000 bootstrap replications. We used iTOL v4.4.2^9^ for graphical modifications and annotations (Letunic and Bork, 2019).

## Results and Discussion

### Experimental Rationale and Strategy

Termite nests maintained for decades at the BAM, offer a unique opportunity to assess a community of microbes, unaffected by abiotic factors. We were inspired by the hypothesis that a stable microbial composition confers protection on a colony and is an indicator of fitness among eusocial insects (Koch and Schmid-Hempel, 2011; Lanan et al., 2016). We compared the microbiomes of three *Coptotermes* species in order to (i) identify the bacterial core microbiome, (ii) analyze the stability of the bacterial community over time, (iii) observe any shifts in the composition when the termite colony was infected by fungi, and (iv) achieve the selective cultivation of underexplored bacterial phyla like Acidobacteria. Our main goal was to screen this resource for underexplored bacterial phyla like Acidobacteria, making them more accessible and thus facilitating the future analysis of their natural products. To this end, we applied a standardized, high-throughput cultivation approach and adapted the conditions to favor the recovery of underexplored and hard-to-cultivate phyla, focusing on the Acidobacteria (Crits-Christoph et al., 2018).

### Bacterial Core Microbiome of Three Domesticated *Coptotermes* spp.

**FIGURE 1:**
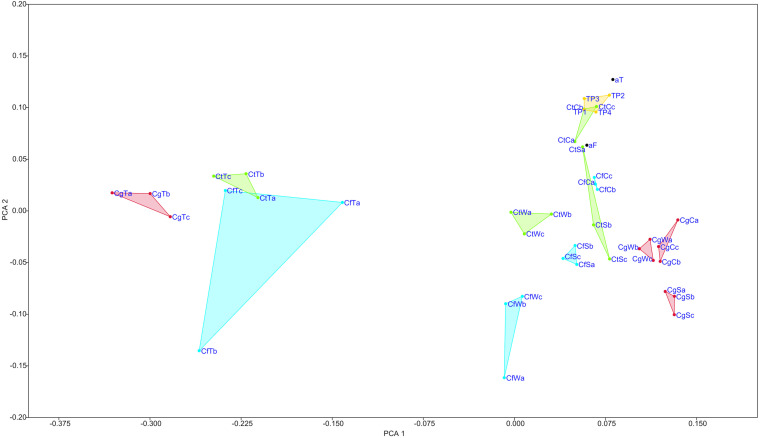
Non-metric multidimensional scaling (nMDS) of all samples. Analysis includes data showing relative abundances of combined replicates from termite microbiomes, their nest material at different levels, cells before and after filtration during Nycodenz density centrifugation (each *n* = 1), and four different time points of *C. testaceus* carton nest material. Triplicates from different nest levels and termites are localized distinctly in the grid. All the *C. testaceus* carton nest samples, analyzed at different time points and filtration steps, form a distinct cluster indicating a similar bacterial composition. TP1, CtC replicate b; TP1–4, sampling time points; Ct, *C. testaceus*; Cg, *C. gestroi*; Cf, *C. formosanus*; C, carton nest material; W, wood material; S, surface; T, termite; aT, after turrax; aF, after 5 μm filtration during Nycodenz density centrifugation. Stress value = 0.124.

To characterize the core microbiomes of three *Coptotermes* species [*C. testaceus* (Ct), *C. formosanus* (Cf) and *C. gestroi* (Cg)] and their nest material, eDNA was isolated from the termites and from different levels of their nests [surface (S), wood (W), and carton nest (C)]. Three samples were taken from each nest level and from corresponding termites. Illumina amplicon sequencing of isolated eDNA yielded 4,724,006 sequences in total, 0.25% of which could not be classified and were defined as *No Relative*. Rarefaction analysis confirmed adequate coverage for statistical calculations (Supplementary Figure 3). Although the rearing temperature and type of wood provided as food differed between Cg and the other species, the termite and nest materials were highly comparable among all three species, with the different nest levels and termites (Figure 1 and Supplementary Figure 4).

Illumina amplicon sequencing revealed that sample *C. testaceus* surface (CtS) featured the greatest number of phylogenetic groups at the genus level (605) and sample *C. gestroi* termite (CgT) featured the least (242) (Table 1). To calculate the ratio between within-group and between-group dissimilarities a one-way ANOSIM was performed including all non-infected samples. No significant differences between sample means were calculated (*p* > 0.2) except the termite group (*p* < 0.01). The Shannon indices were similar among the samples of nest material (2.9–3.7) and among the termites (2.3–2.6), but the termite samples showed lower overall microbial diversity (Table 1). This indicates that the termite samples inhabit a narrower selection of microbes than the surrounding nest material, probably reflecting selection for exo- and endosymbionts with specific metabolic roles (Brune and Dietrich, 2015).

**TABLE 1:** Richness and diversity indices based on amplicon sequencing data from termite and termite nest samples.

**Termite species**	**Level**	**Number of sequences^a^**	**Number of phylogenetic groups^b^**	**Simpson^c^**	**Shannon^d^**	**Evenness^e^**	**Chao 1^f^**
*C. gestroi*	CgS	322,114	549	0.92	3.7	0.05	245
	CgW	318,647	549	0.93	3.6	0.06	221
	CgC	309,675	392	0.93	3.5	0.06	187
	CgT	210,185	242	0.78	2.3	0.03	128
*C. testaceus*	CtS	896,649	605	0.92	3.0	0.03	232
	CtW	218,641	432	0.95	3.6	0.06	146
	CtC	450,553	404	0.89	2.9	0.04	222
	CtT	306,701	260	0.68	2.3	0.03	125
*C. formosanus*	CfS	257,399	444	0.91	3.3	0.04	220
	CfW	231,098	392	0.91	3.2	0.05	212
	CfC	217,688	279	0.90	3.0	0.05	136
	CfT	174,613	277	0.75	2.6	0.04	150
Nycodenz	aT	139,879	241	0.85	2.3	0.06	169
	aF	245,690	438	0.90	3.0	0.05	110
Sampling time points	TP1	105,574	217	0.85	3.1	0.05	132
	TP2	35,320	204	0.90	2.4	0.05	196
	TP3	95,298	242	0.86	2.8	0.05	232
	TP4	53,558	275	0.89	2.6	0.05	152
*C. testaceus* nest material	eS	34,709	442	0.97	4.2	0.20	325
facing fungal burden	lS	100,015	483	0.90	3.4	0.12	238

*Replicates of nest materials and termites were combined for analysis. Statistical analysis was performed using PAST v3.18. Sample abbreviations: Ct, C. testaceus; Cg, C. gestroi; Cf, C. formosanus; C, carton nest material; W, wood material; S, surface; T, termite; TP, time point; aT, before 5 μm filtration; aF, after 5 μm filtration during Nycodenz density centrifugation; eS, early stage of fungal infection; lS, late stage of fungal infection. ^*a*^Total number of sequences per sample derived from Illumina sequencing. ^*b*^Total number of different phylogenetic groups in each sample. ^*c*^Simpson index represents the diversity. If the value is 0, diversity is at zero level, whereas 1 is the highest. ^*d*^Shannon describes the diversity of the data and considers the abundance and number of phylogenetic groups. ^*e*^Evenness represents the equal distribution of phylogenetic groups per sample: 0 = equal and 1 = unequal distribution. ^*f*^Estimated species richness of each sample.*

**FIGURE 2:**
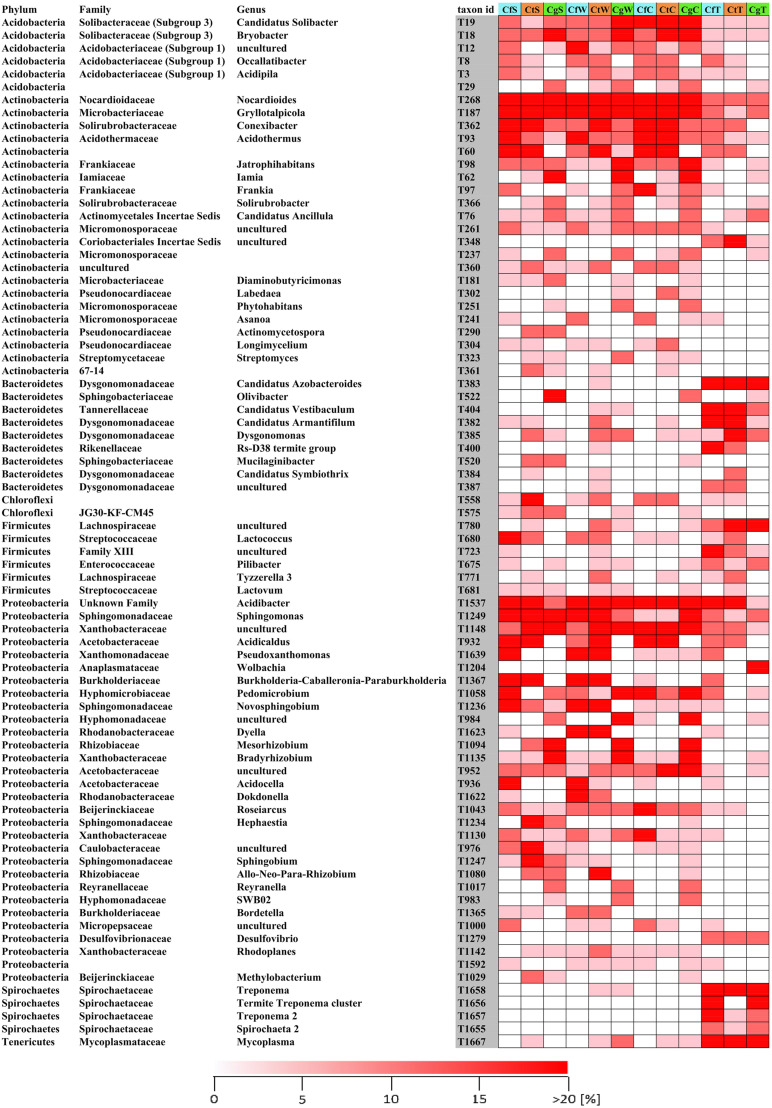
Relative abundances of the 80 most abundant phylogenetic groups. Samples of nest material were collected from the surface, wood and carton nest (*n* = 3), and corresponding termites (*n* ≥ 12–23) of three species (*C. formosanus*, *C. gestroi*, and *C. testaceus*). If the assignment of phylogenetic groups to the genus level was not possible it was left blank. Replicates were combined for visualization. Abundances indicate microbial communities shared among all nest levels or associated with termites. Ct, *C. testaceus*; Cg, *C. gestroi*; Cf, *C. formosanus*; C, carton nest material; W, wood material; S, surface; T, termite.

In all termite samples, Bacteriodetes was the most abundant phylum (52%), followed by Alphaproteobacteria (12%), Spirochetes (11%), Firmicutes (7%), and Actinobacteria (6%) (Figure 2). Compared to the captive termites of this study, those phyla were already observed in comparable abundances in free-living *C. gestroi* harvested in Vietnam (Do et al., 2014) and *C. testaceus (formerly C. niger)* (Dietrich et al., 2014) as well as *C. curvignathus* guts *from Malaysia* (King et al., 2014). Particularly, the genera *Alistipes*, *Desulfovibrio*, *Treponema*, *Dysgonomonas*, *Mycoplasma*, and *Burkholderia* (Supplementary Table S1) and order Actinomycetales, Xanthomonadales, Pseudomonades (Figure 2) were also described as highly abundant in other studies (Brune and Dietrich, 2015)*;* Among the Bacteroidetes, the order Bacteroidia was most abundant (up to >98%), whereas Candidatus Azobacteroides represented the most abundant genus (>50%) (Supplementary Table S1). Some Bacteroidia are known as obligate intracellular symbionts that live within cellulolytic protists in the termite gut (Hongoh et al., 2008). They are essential for nitrogen fixation and the degradation of complex carbon sources, but are not particularly enriched in natural product gene clusters (Stingl et al., 2004; Sun et al., 2015).

**FIGURE 3:**
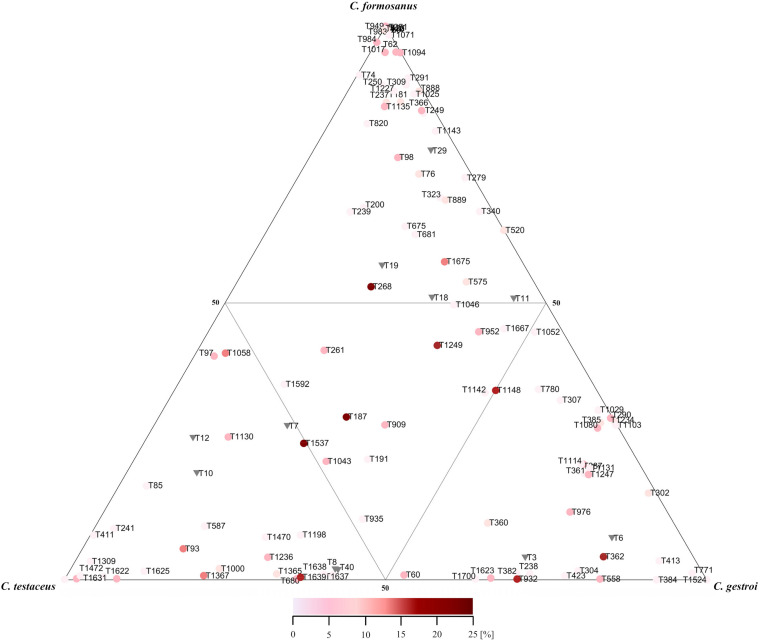
Ternary plot of the relative abundances of shared bacterial phylogenetic groups among all nest materials of three domesticated *Coptotermes* species. Mean values of replicates (*n* = 3) from each nest level were combined. Only phylogenetic groups with a relative abundance >0.1% in at least one nest were included in the analysis. Colors indicate the relative abundance per sample of each phylogenetic group as indicated by the scale bar. Shared phylogenetic groups with the same abundance appear in the inner triangle. Phylogenetic groups belonging to the phylum Acidobacteria are marked with inverse triangles. An assignment of each phylogenetic group is provided in Supplementary Table S1.

In all nest samples, the most abundant phyla were Actinobacteria, Alphaproteobacteria, Gammaproteobacteria and Acidobacteria, which together represented more than 90% of all phylogenetic groups (Supplementary Figure 4). The most abundant genera/families were present in all nest samples from all three *Coptotermes* species, and did not change significantly over time. Ranked by relative abundance based on our heat map and ternary plot, the most prevalent groups were *Conexibacter*, uncultivated Xanthobacteraceae, *Acidicaldus*, *Acidibacter*, *Acidothermus*, *Sphingomonas*, uncultivated Acetobacteraceae, *Roseiarcus*, *Occallatibacter*, *Candidatus Solibacter*, *Bryobacter*, *Xiphimenatobacter*, *Nocardioides*, *Gryllotalpicola*, and *Frankia* (Figures 2, 3). We therefore propose, that these taxonomic genera are part of the core microbiome.

Although the phylum Actinobacteria was the most abundant in our nest samples, the classical members associated with natural product synthesis were rare [mean < 0.5%, standard error (SE) = 0.01]. In particular, streptomycetes were ∼10-fold underrepresented (<0.15%, *SE* = 0.12) compared to soil habitats (Zhang et al., 2019). To exclude the possibility that spore-forming actinomycetes were under-detected due to an experimental bias in eDNA isolation, the eDNA extraction method was evaluated using an actinomycete spore suspension. This confirmed the detection of actinomycete DNA and ruled out the artifactual exclusion of DNA from spore-forming bacteria (data not shown). Furthermore, nMDS analysis revealed overlaps in the composition of the surface, wood and carton nest microbiomes, suggesting that microbes are transferred within the nest. Compared to the other samples, the carton nest showed the least diverse bacterial microbiome, indicating a specialized bacterial composition (Figure 1 and Table 1).

**FIGURE 4:**
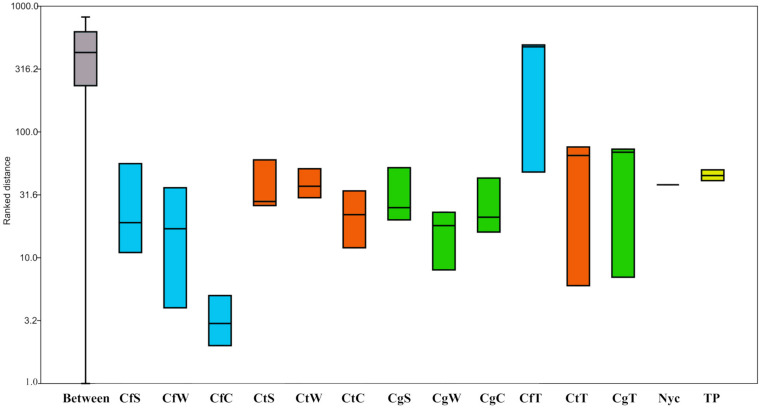
Analysis of similarities (ANOSIM) of all nest materials taken from three captive *Coptotermes* species. One-way ANOSIM shows the ratio between within-group and between-group dissimilarities. Replicates of each sample, time point, and step (before and after filtration) were grouped together for analysis. PAST v3.18 was used for the calculations based on the Bray-Curtis similarity index. Box-plots were calculated using the interpolated quartile method. No significant differences between two or more groups exist except the termite group (*p* < 0.01). Cf, *C. formosanus* (blue); Ct, *C. testaceus* (orange); Cg, *C. gestroi* (green); C, carton nest material; W, wood material; S, surface; T, termites; Nyc, before and after 5 μm filtration; TP, four different sampling time points for the *C. testaceus* carton nest (yellow).

The total proportion of Acidobacteria in all nest materials (mean = 6.4%, *SE* = 2.3) was higher than in the termites (mean = 1.5%, *SE* = 1.99). For example, the abundance of Acidobacteria in sample CtC (7.8%, *SE* = 2.2) was greater than in sample CtT (2.7%, *SE* = 2.8) (Supplementary Figure 4). The Acidobacteria in the nest material were also more diverse than those in the termites. The major subgroups of Acidobacteria present in CtC were subgroups 1 (35%), 3 (50%), and 4 (11%), whereas 2% of the reads could not be assigned to a specific subgroup (Supplementary Figure 5). Comparison of the termite and nest material samples revealed clearly distinguishable dissimilarities (Figures 1, 4).

Taken together, these experiments showed that all nest materials had similar diversity indices, indicating that the material was exposed to limited external influences and allowed a diverse microbial community to flourish. The taxonomic diversity of Acidobacteria (in terms of different subdivisions) was not greatly enhanced in CtC compared to the other nest materials, however, their nest material was selected for further investigation, because the statistical analysis and the abundance of Acidobacteria (7.8%, compared to a mean of 6.0%) made it a promising source for the isolation and cultivation of strains representing this phylum.

### Stability of the *C. testaceus* Carton Nest Microbiome

Abiotic factors such as aeration, temperature and humidity, which are regulated by the architecture of termite nests, play a key role in microbiome stability (Marins et al., 2016). Among the various nest compartments, the carton nest is expected to contain the most adapted microbiota because it consists of salivary secretions and feces (Enagbonma et al., 2020). Furthermore, the carton nest is frequented by termites and protected from external contact except during feeding. We used the carton nest material as an accessible resource that enables routine sampling and the generation of comparative datasets over time. Therefore, we first investigated the temporal stability of the microbial community.

The *C. testaceus* carton nest material was assessed at four time points over a period of 2 years to determine the comparability and statistical robustness of the amplicon sequencing data (Figures 1, 4). Krona pie charts were constructed to compare the composition of the microbiomes over time (Supplementary Figure 6). The most abundant families were ranked as follows: Solirubrobacteraceae, Acetobacteraceae, Xanthobacteraceae, an unknown family of the class Gammaproteobacteria, Solibacteraceae, Acidobacteriaceae, Xipinematobacteraceae, Gemmataceae, Microbacteriaceae, Nocardioidaceae, IMCC26256 and Acidotermaceae. The stability of this microbial community was confirmed by their mean species evenness of 0.04 (*SE* = 0.01), their Shannon indices (Table 1), their nMDS scores (Figure 1), and the absence of significant changes identified by ANOSIM (*p* > 0.05, *R* < 0.35). The stability of the community is likely to be promoted by the unchanging abiotic and biotic conditions in the carton nest, including the constant temperature, humidity and light, and the consistent diet, which determines the activity of termites and the content of their secretions and excretions. The remarkable stability of the microbiome over time ensured that Acidobacteria were constantly present in the carton nest material.

### Relationship Between Microbiome Stability and Termite Fitness

**FIGURE 5:**
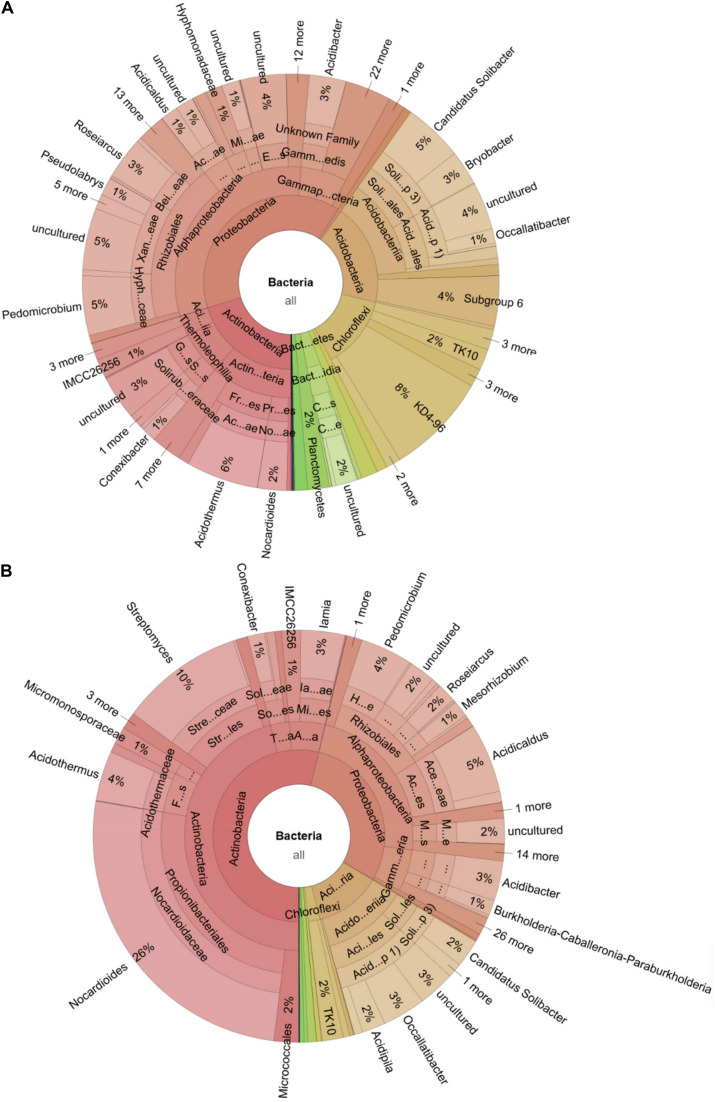
Microbiome shift during the spreading of a fungal infection in a *C. testaceus* carton nest. The Krona chart shows the microbiome of the infected nest material during **(A)** the early and **(B)** the late stage of infection. The abundance of Acidobacteria and Chloroflexi during the early stage of infection was significantly higher compared to a healthy nest microbiome. The abundance of *Streptomyces* (early 0.03–10% late), Acidobacteria (early 19–12% late) and *Nocardioides* (early 2–26% late) was higher compared to the healthy nest material. The abundance of phylum Chloroflexi decreased from 14 to 2% from the early to the late infection stage.

To determine whether the health status of termite populations has an effect on the stability of the microbiome, we studied a second *C. testaceus* nest at this time, which unintentionally suffered from a fungal infection that became progressively more severe between sampling intervals (Supplementary Figure 7). Over time, the number of individual termites in the colony declined, ultimately to zero. No live specimens or even corpses were recovered from the nest material facing a fungal burden at the final sampling point. The microbiome of soldier termites retrieved from non-infected and infected showed little change during the early stages of infection (Supplementary Figure 8). In contrast, there was a remarkable shift in the composition of the nest material microbiome, with particular shifts in the abundance of Chloroflexi, Acidobacteria and the genus *Streptomyces* (Figure 5) compared to healthy nest material (Supplementary Figure 4). The abundance of *Streptomyces* spp. increased by more than 30-fold between the early and late stages of infection, which has been reported before in *C. formosanus* nests due to opportunistic mutualism between the termites and *Streptomyces* spp. with antifungal activity (Chouvenc et al., 2013, 2018). We also found that, during the early stage of infection, Acidobacteria were twice as abundant compared to uninfected nests (Figure 5 and Supplementary Figure 4). Accordingly, we surveyed Acidobacteria in the different *C. testaceus* nest compartments. The carton nest showed generally broad taxonomic diversity and was enriched for Acidobacteria and other underexplored phyla, such as members of the PVC superphylum (Wagner and Horn, 2006), which accounted for more than 10% of the bacterial consortium (Supplementary Figure 4). Genome and metagenome analysis have shown that underexplored phyla such as Acidobacteria also carry biosynthetic gene clusters for the synthesis of specialized metabolites such as modified ribosomal peptides (Skinnider et al., 2016; Eichorst et al., 2018), polyketides, and non-ribosomal peptides (Crits-Christoph et al., 2018).

The comparative analysis of these gene clusters has shown a remarkable degree of genetic diversity compared to well-characterized gene clusters in other bacteria (Crits-Christoph et al., 2018). The genetic divergence among these clusters is often correlated with structural diversity of the corresponding secondary metabolites, providing confidence that such bacteria may lead to the discovery of new chemical entities (Medema et al., 2014). The opportunity to find gene clusters for secondary metabolism increases in bacteria with genome sizes exceeding 8 Mb (Baltz, 2017). The largest genome reported for a cultivated member of the phylum Acidobacteria is 9.9 Mb (Ward et al., 2009), whereas the mean size of the 23 published type strains of Acidobacteria^10^ genomes is ∼5.2 Mb. Furthermore, horizontal gene transfer has been reported among the Acidobacteria, which indicates a potential to acquire functions with a defensive or regulatory advantage in their environment (Challacombe et al., 2011). For example, chitinolytic and cellulolytic activities are widespread among the Acidobacteria, which would be useful for the exploitation of nutritional niches in termite nests (Kielak et al., 2016; Belova et al., 2018). This may also benefit termites facing infections, as already reported for *Streptomyces* spp. associated with termite nest material (Carr et al., 2012; Chouvenc et al., 2018; Klassen et al., 2019). Although, Acidobacteria is the most abundant phylum in some habitats (Chan et al., 2006; Weijers et al., 2009), fewer than 60 Acidobacteria strains have been cultivated thus far (Kielak et al., 2016), whereas four classes have been proposed with validly published names^11^. Most of the isolates belong to subgroup 1, but still the number of available strains is only ∼20 (Damste et al., 2017). A larger number of Acidobacteria strains with broader diversity must be made available in order to exploit their genetic and metabolic repertoire for natural product discovery. Given that the abundance of Acidobacteria doubled to 19% during the early stages of the fungal infection and that they remained more abundant (12%) than *Streptomyces* spp. (10%) even at the later stage (Figure 5), we sought to increase their general accessibility by a combination of targeted and high-throughput cultivation.

### Targeted Cultivation and Phylogenetic Classification of Enriched Acidobacteria

**FIGURE 6:**
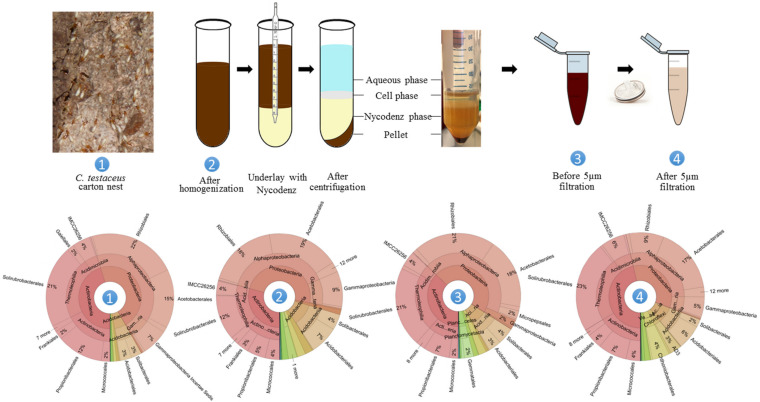
Retrieval of living bacteria from *C. testaceus* carton nest samples. The microbiome composition was analyzed at four key steps of the Nycodenz density gradient centrifugation method. No bias in bacterial diversity was observed. Filtration using a 5-μm nylon syringe filter was necessary to remove particles for subsequent cell counting. The statistical comparison of these data with other data from this study can be found in Figures 1, 4. Adapted with permission (Barra Caracciolo et al., 2005).

The core bacterial community of the healthy *C. testaceus* carton nest material remained stable for 2 years. It showed broad taxonomic diversity but was enriched for Acidobacteria, and this phylum was further enriched by fungal infection. We chose the healthy *C. testaceus* carton nest material as the source for our targeted cultivation process to circumvent the fungal bias. To enable high-throughput cultivation, we used a small-scale microplate format. Thus, cells were retrieved based on Nycodenz density centrifugation used to enrich cells from complex environmental matrices, therefore, to enable subsequent cell enumeration via flow cytometry. Previous reports have indicated that using Nycodenz density centrifugation for soil samples can introduce bias (Holmsgaard et al., 2011). However, this may reflect the sample origin, given that the same method did not affect cell viability, or the distribution and proportion of the microbial community, during the analysis of fecal samples (Hevia et al., 2015). Accordingly, we first evaluated our adapted method to determine whether the retrieval of living cells from termite nest matrices influenced the composition of the bacterial community. We compared crude nest material with a homogenate and with cell layers before and after filtration (Figure 6). Statistical analysis using ANOSIM of the amplicon data revealed no significant shift in the bacterial community (*p* > 0.1), which was supported by bacterial composition analysis (Figure 6). Furthermore, nMDS analysis confirmed that the pivotal steps before and after filtration in the Nycodenz gradient centrifugation protocol are similar compared to the *C. testaceus* carton nest samples (Figure 1). The material was passed through a 5-μm syringe filter to separate cells from residual matrix particles. This is necessary for accurate cell counting by FACS, otherwise particles of the nest matrix can produce background noise. Following this treatment, the composition of the bacterial community (Supplementary Figure 4), the Shannon index (Table 1), and the ANOSIM and nMDS clustering profiles, indicated no remarkable changes (Figure 1). Indeed, the cell suspension derived from the Nycodenz and filtration steps were similar to the untreated nest material samples of *C. testaceus* (aF and aT, Figure 1). This confirms that the procedure we used was able to extract living cells from termite nest material with negligible experimental bias, and our target phylum was reliably extracted from the matrix (Figure 6). In the next step, cells were counted by staining with SYTO9 followed by analysis using flow cytometry, which indicated a total cell concentration (living and dead) of ∼8 × 10^8^ cells/mL (Supplementary Figure 9).

Based on earlier studies concerning the success of cultivating bacteria from environmental samples (Epstein, 2013; Ling et al., 2015), and taking the live/dead ratio (∼70:30) into account, we estimated that ∼40 cells should be distributed into each well of a 384-well plate containing 40 μL of medium per well. This was based on the assumption that only a small fraction would be able to grow in the synthetic VL55 medium (pH 5.5) supplemented with xylan as the only carbon source, a medium already shown to be suitable for the cultivation of Acidobacteria (Campanharo et al., 2016). This medium suppresses the growth of bacteria that cannot grow on complex carbon sources at low pH, and therefore favors the growth of the majority of Acidobacteria (Belova et al., 2018; de Chaves et al., 2019). Furthermore, Acidobacteria appear to thrive when presented with a lower concentration of trace elements than normally found in complex media (de Chaves et al., 2019). An additional advantage of the process described here is its ability to propagate the cultures quickly in order to reduce the risk of competition from faster-growing bacteria that take longer to become established due to the chosen medium, such as certain Proteobacteria (George et al., 2011; Campanharo et al., 2016).

Following the pipeline, 4,291 wells in total were re-inoculated from 384- to 96-deepwell microplates. After seven days of incubation, 4,028 wells were determined as grown via turbidimetry (93.9%). In total, 3,456 individual cultures (85.8%) were successfully sequenced via 16*S* rRNA gene sequencing (Sanger method, using the reverse primer only). Among these, 235 wells (6.8%) were affiliated with the target phylum Acidobacteria. After reduction for redundancy—based on 16*S* rRNA sequencing (Sanger method, using forward and reverse primer)—the unique wells were propagated onto agar plates. Finally, four unique strains were isolated. However, only three could be propagated furthermore and were therefore integrated into our strain collection with the IDs FhG110202, FhG110206, and FhG110214.

**FIGURE 7:**
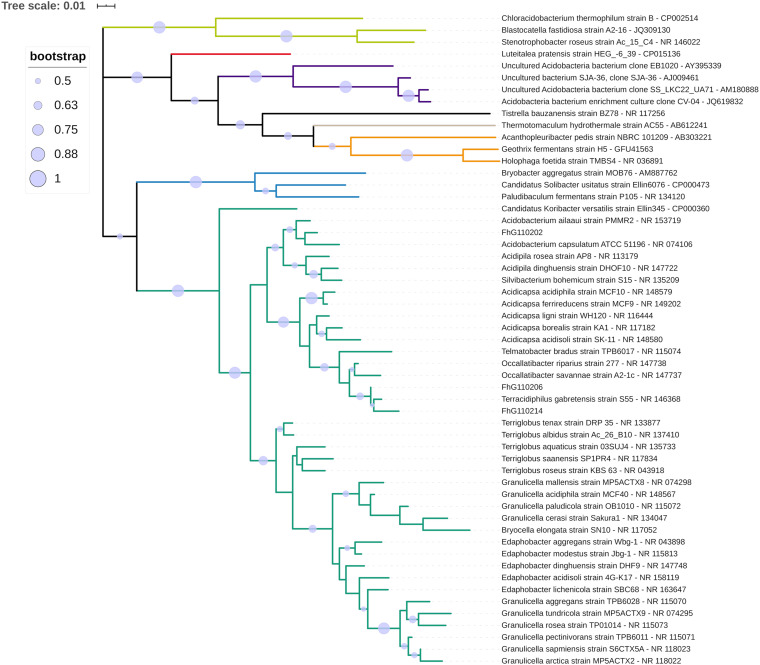
Phylogenetic classification of the isolated strains FhG110202, FhG110206, and FhG110214 within the phylum Acidobacteria. The tree is based on a ClustalW alignment of available 16*S* rRNA gene sequences between positions 113 and 1,357 [based on *Escherichia coli* 16*S* rRNA gene numbering (Brosius et al., 1978)] from the 40 most similar sequences to the reference strain FhG110202, and also includes FhG110206, FhG110214, and representatives of Acidobacteria subgroups 3, 4, 6, 7, 8, and 10. The tree was calculated using MEGA v7.0.26 with the maximum-likelihood method and Tamura-Nei model. Circles on the tree branches indicate bootstrap values of 1,000 bootstrap replicates with a bootstrap support of more than 50%. Subgroup affiliations are indicated by colors. The new isolates are indicated by a green arrow. The tree is drawn to scale, with branch lengths measured in the number of substitutions per site.

The phylogenetic classification of the three strains within the phylum Acidobacteria based on nearly full-length 16*S* rRNA gene sequences revealed their assignment to three different genera within subgroup 1 (Figure 7). FhG110202 was most closely related to the type strain of *Acidobacterium ailaaui* (97.7%), a bacterium that was isolated from a geothermally active microbial mat on Hawaii (Myers and King, 2016). FhG110214 was most closely related to *Terracidiphilus gabretensis* (97.1%), which was isolated from a boreal forest in a Czech national park (Garcia-Fraile et al., 2016). FhG110206 was most closely related to *T. gabretensis* (98.9%), *Occalibacter riparius* (98.4%), and FhG110214 (97.9%). A sequence identity threshold of 98.65% indicates species differentiation (Kim et al., 2014). The sequence identity between the 16*S* rRNA genes of FhG110214 and strain of *T. gabretensis* S55^T^ (97.1%), and FhG110202 and strain of *A. ailaaui* PMMR2T^T^ (97.7% identity), suggests that further experiments should be carried out to determine whether those strains represent new species.

Further investigations should also be carried out to determine the beneficial role of Acidobacteria for xylophagous *Coptotermes* spp. in terms of their potential to produce natural products cohering with their enrichment during the early and late stages of a fungal infection such as *Streptomycetes*. Our work therefore adds to the number of strains from the interesting phylum of Acidobacteria that are available for further analysis.

## Conclusion

In this study, we evaluated the use of laboratory-bred termite colonies (*Coptotermes* spp.) and their nest materials as bioresources for the isolation of underexplored Acidobacteria, which are currently difficult to access or cultivate with sufficient diversity for bioprospecting. This is a key requirement because the likelihood of discovering new chemical entities is thought to be higher in underexplored phyla compared to classical phylogenetic groups that are already known to synthesize natural products. Our in-depth analysis at the genus level of microbial communities associated with three different termite species revealed the carton nest as the best source of Acidobacteria, with a 10-fold enrichment compared to the termites themselves. The microbial community showed high temporal stability in the healthy colony but underwent a profound shift during the late stage of a fungal infection favoring the proliferation of *Streptomyces* spp. and Acidobacteria. In summary, we applied a high-throughput cultivation process adapted to the metabolic repertoire of Acidobacteria. This led to the successful isolation of three novel strains of Acidobacteria, which may shed light on their biological relationship with xylophagous lower termites due to their accessibility for natural product discovery.

## Data Availability Statement

The datasets generated for this study can be found in the following online repositories. The data can be accessed under the BioProject PRJNA657759 and the 16S rRNA sequences can be accessed under following numbers: MT895693, MT898545, and MT895791 (https://www.ncbi.nlm.nih.gov/genbank/).

## Author Contributions

MO, MS, and JG conceived and designed the experiments. JG initiated the isolation of Acidobacteria. MO, CZ, TC, MS, BL, and SM contributed to the cultivation and isolation experiments. MO, CZ, TS, JG, and SG analyzed the data. BL and MO implemented the liquid handling processes. MO drafted the first manuscript. RP and DM were responsible for the rearing, supply of termites, and revised the manuscript. MO, TC, JG, and RP performed sampling campaigns. TS, PH, and AV organized the manuscript writing. JG and TS supervised the research, helped to draft the manuscript, and revised it. AV acquired funding from the state of Hesse. AV and PH initiated the public-private partnership between Fraunhofer and Sanofi. All authors contributed to the article and approved the submitted version.

## Conflict of Interest

The authors declare that the research was conducted in the absence of any commercial or financial relationships that could be construed as a potential conflict of interest.

## Footnotes

1 http://bbmap.sourceforge.net

2 http://www.bioinformatics.org/cd-hit

3 http://www.arb-silva-de

4 http://blast.ncbi.nlm.nih.gov/Blast.cgi

5 https://github.com/marbl/Krona/wiki

6 https://folk.uio.no/ohammer/past/

7 https://www.ncbi.nlm.nih.gov/

8 https://www.megasoftware.net

9 https://itol.embl.de/

10 https://www.ezbiocloud.net/search?tn=acidobacteria

11 https://lpsn.dsmz.de/phylum/acidobacteria

## Supplementary Material

Supplementary Table 1 Taxonomic classification and the respective calculated relative abundance using Illumina amplicon sequencing data and additionally the SILVAngs v1.3 analysis pipeline.
