# Supplementary material for: High-Throughput Cultivation for the Selective Isolation of Acidobacteria From Termite Nests
Source: Front Microbiol. 2020 Nov 6;11:597628. doi: 10.3389/fmicb.2020.597628 (PMC7677567; doi:10.3389/fmicb.2020.597628)
Supplement: Supplementary file 2 [file Data_Sheet_1.pdf]

Supplementary material

High-throughput cultivation for the selective isolation of Acidobacteria from termite nests

Markus Oberpaul[1], Celine M. Zumkeller[1], Tanja Culver[1], Marius Spohn[1], Sanja Mihajlovic[1], Benedikt Leis[1], Stefanie Glaeser[2], Rudy Plarre[3], Dino P. McMahon[3,4], Peter Hammann[5†], Till F. Schäberle[1,6]*, Jens Glaeser[1†]* and Andreas Vilcinskas[1,6]*

[1] Fraunhofer Institute for Molecular Biology and Applied Ecology (IME), Branch for Bioresources, Giessen, Germany

[2] Institute of Applied Microbiology, Justus Liebig University Giessen, Giessen, Germany

[3] Bundesanstalt für Materialforschung und -prüfung, Berlin, Germany

[4] Institute of Biology, Free University of Berlin, Berlin, Germany

[5] Sanofi-Aventis Deutschland GmbH, R&D Integrated Drug Discovery, Hoechst industrial park, Frankfurt am Main, Germany

[6] Institute for Insect Biotechnology, Justus Liebig University Giessen, Giessen, Germany

*** Correspondence:**
PD Dr. Jens Glaeser
Jens.glaeser@evotec.com

Prof. Dr. Till F. Schäberle
Till.F.Schaeberle@agrar.uni-giessen.de

Prof. Dr. Andreas Vilcinskas
Andreas.Vilcinskas@agrar.uni-giessen.de

† Present addresses:
Peter Hammann and Jens Glaeser: Evotec International GmbH, Göttingen, Germany

Keywords: termites, *Coptotermes*, core microbiome, natural products, Acidobacteria, underexplored phyla, social insects, termite-associated microbes

1 Supplementary figures

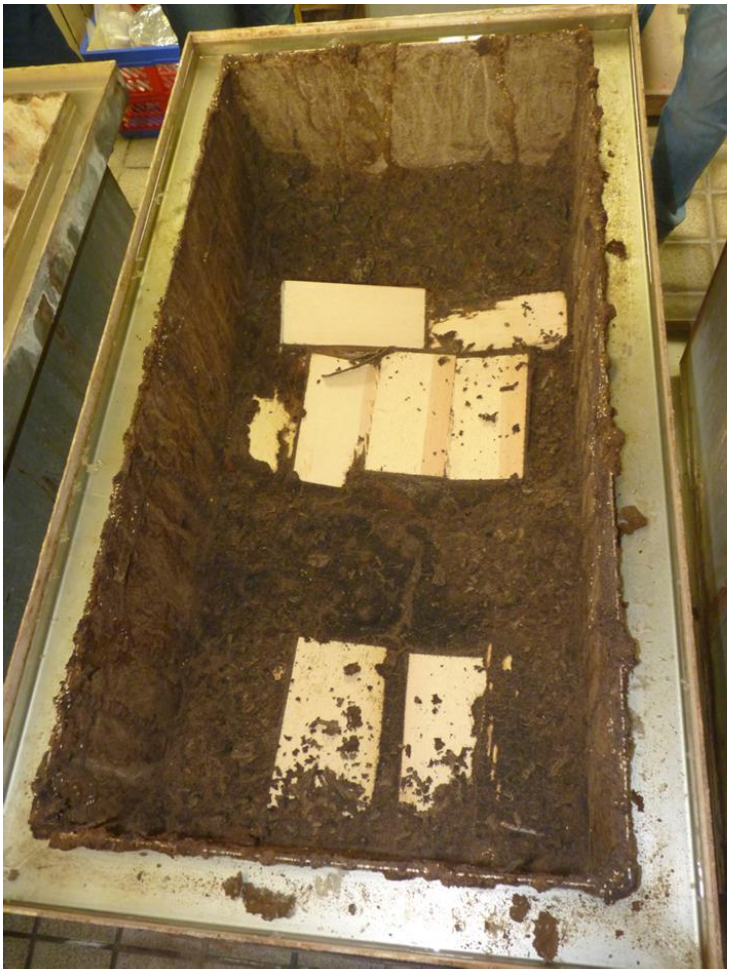

Supplementary Figure 1. Rearing box for *Coptotermes* species located at the Bundesanstalt für Materialforschung und -prüfung (BAM) in Berlin. Wood pieces for breeding were directly placed onto the nest mound. A trap filled with water surrounding the nest prevents termites from breaking out.

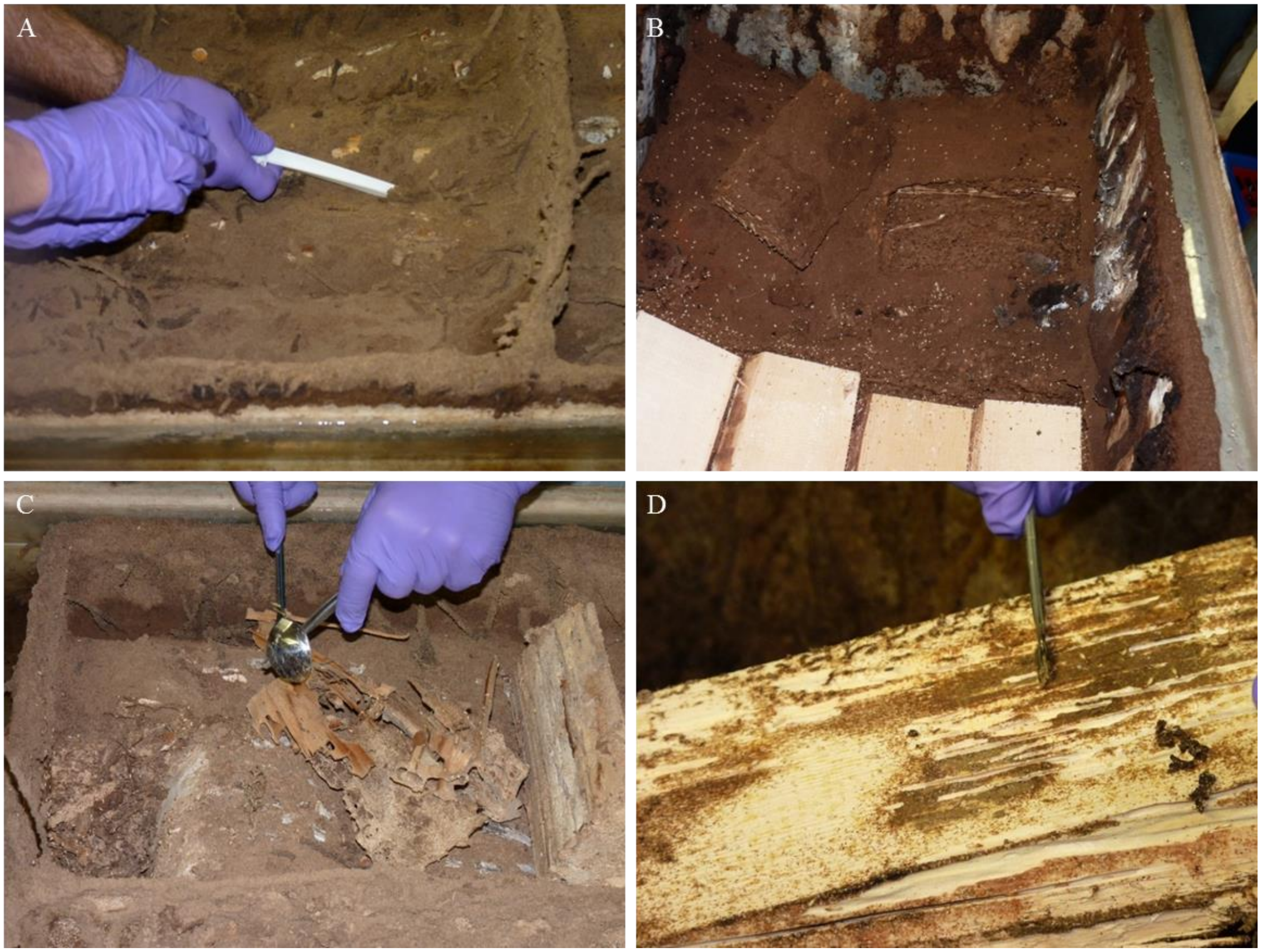

Supplementary Figure 2. Sampling of termite nest material. Nest material from the surface was collected with sterile spatula (*surface*) (**A**). Nest material and wood were excavated in order to sample carton nest material (*carton nest*) (**B**). Old pitted wood pieces were collected to retrieve the cells from those samples (*wood*) (**C**). Wood pieces from feeding events were placed aside and biofilms were sampled from the fresh eroded base and combined with the old pitted pieces (*wood*) (**D**).

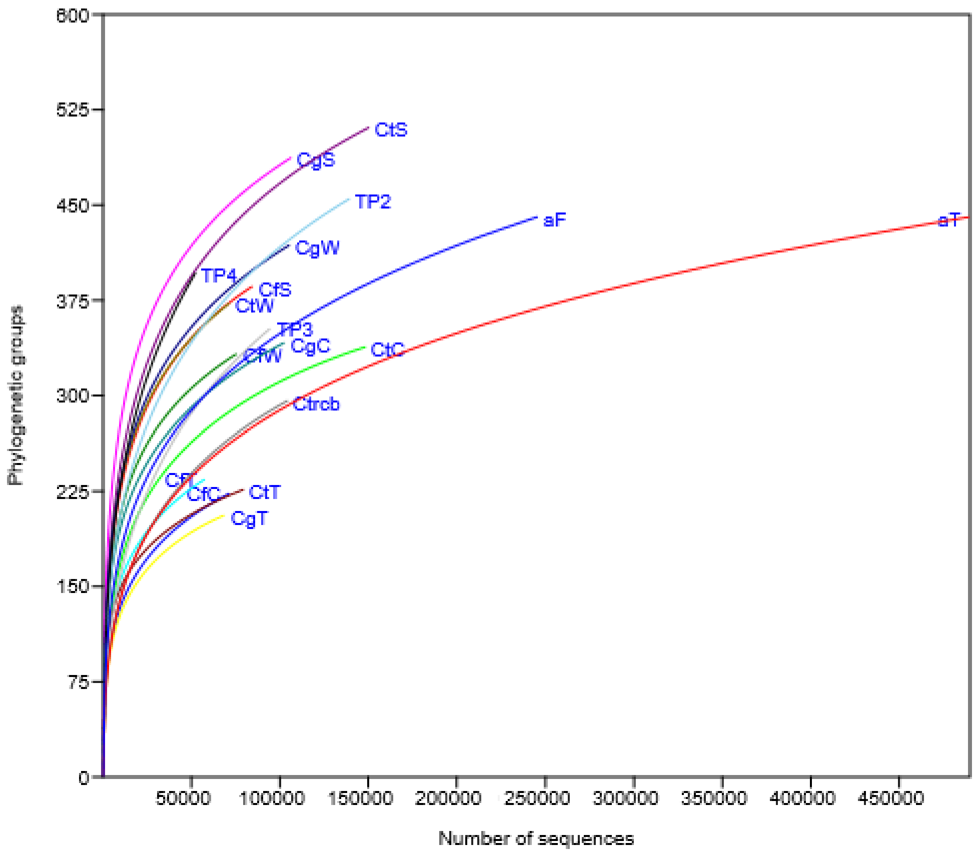

Supplementary Figure 3. Rarefaction curves (number of sequences vs. phylogenetic groups) of median of triplicates *Coptotermes spp.* nest material, before and after filtration and 4 time points based on Illumina 16*S* rRNA gene amplicon sequencing data. TP1–4 = represents one of each time point. aT = before 5 µm filtration; aF = after 5 µm filtration of cell fraction received by the Nycodenz density gradient centrifugation; Ct = *C. testaceus*; Cg = *C. gestroi*; Cf = *C. formosanus*; C = carton nest material; W = wood material; S = surface; T = termites.

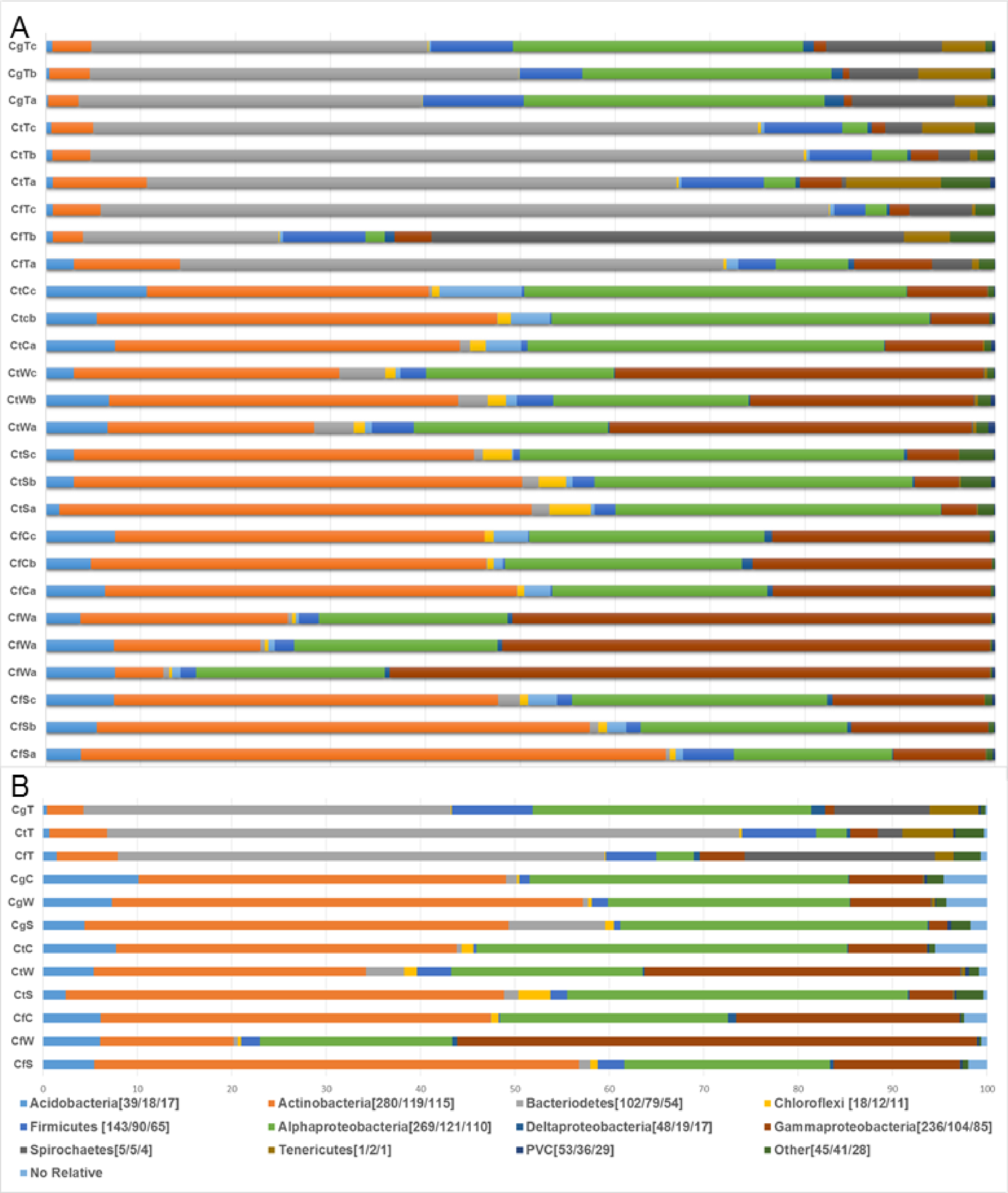

Supplementary Figure 4. Relative abundances of bacteria on phylum level of uninfected nest material and termites. Triplicates are indicated by a, b and c. CfTb can be identified as outlier (**A**). Mean values of the three replicas of (A) added together (**B**). Numbers in square brackets indicate the number of phylogenetic groups found within the corresponding phylum, respectively [total no. of phylogenetic groups in nest material / total no. of phylogenetic groups in termites / no. of shared phylogenetic groups].

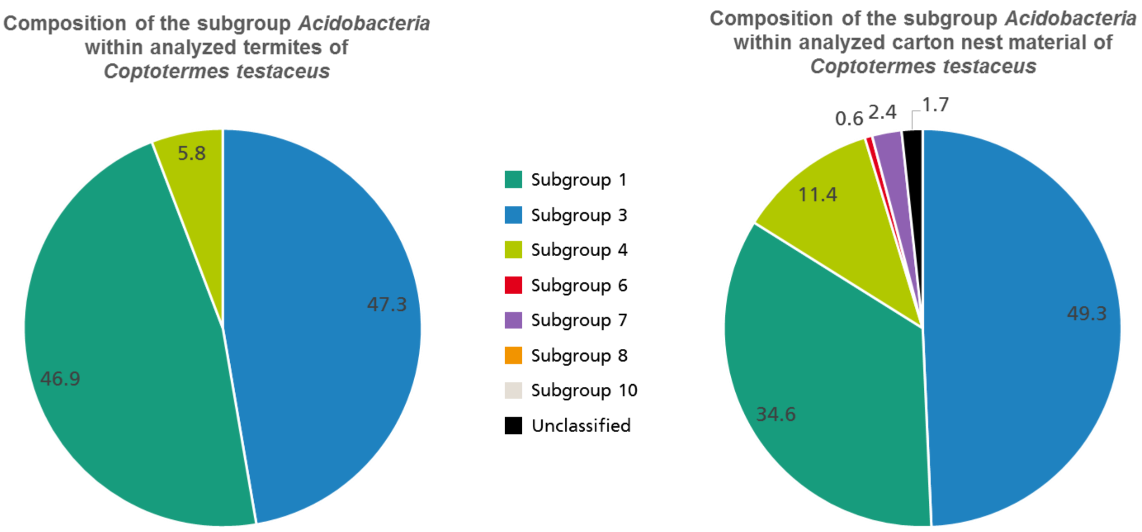

Supplementary Figure 5. Pie chart illustrating the subgroup of *Acidobacteria* composition within *C. testaceus* individuals (left) and the respective carton nest (right). The subgroup classification is based on the best blast hit including alignment length and SILVA database annotation. Subgroup 3 is most abundant in the carton nest material of *C. testaceus* (49.3%), followed by subgroup 1 (34.6%) and subgroup 4 (11.4%). A minor fraction of 4.7% is divided into subgroup 6 (0.6%), Subgroup 7 (2.4%) and an unclassified group of 1.7%. The total abundance of *Acidobacteria* in CtC, as well as the diversity is higher in their carton nest (7.8%) compared to the termites (0.63%). In the latter, only subgroups 1 (46.9%), 3 (47.3%) and subgroup 4 (5.8%) were detected.

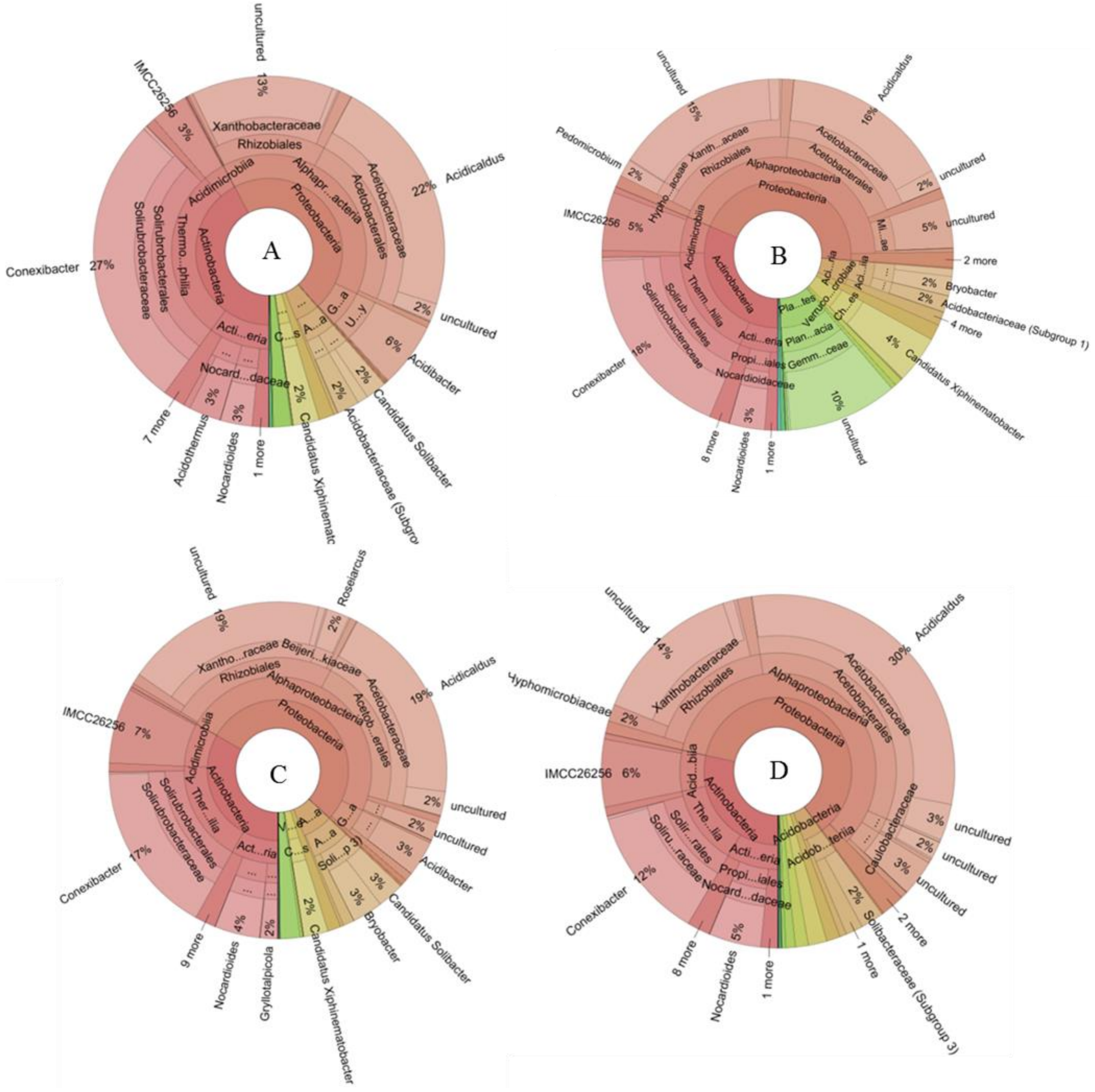

Supplementary Figure 6. Krona pie charts displaying the relative abundances of bacterial composition on family level sampled at four time points over two years. The most abundant families in the carton nest of *C. testaceus* were Solirubrobacteraceae, Acetobacteraceae, Xanthobacteraceae, an unknown family of the class Gammaproteobacteria, Solibacteraceae, Acidobacteriaceae, Xipinematobacteraceae, Gemmataceae, Microbacteriaceae, Nocardioidaceae, IMCC26256 and Acidotermaceae. Evenness average = 0.004; Standard error = 0.01. Data indicate a stable bacterial composition in the carton nest of *C. testaceus* over 2 years.

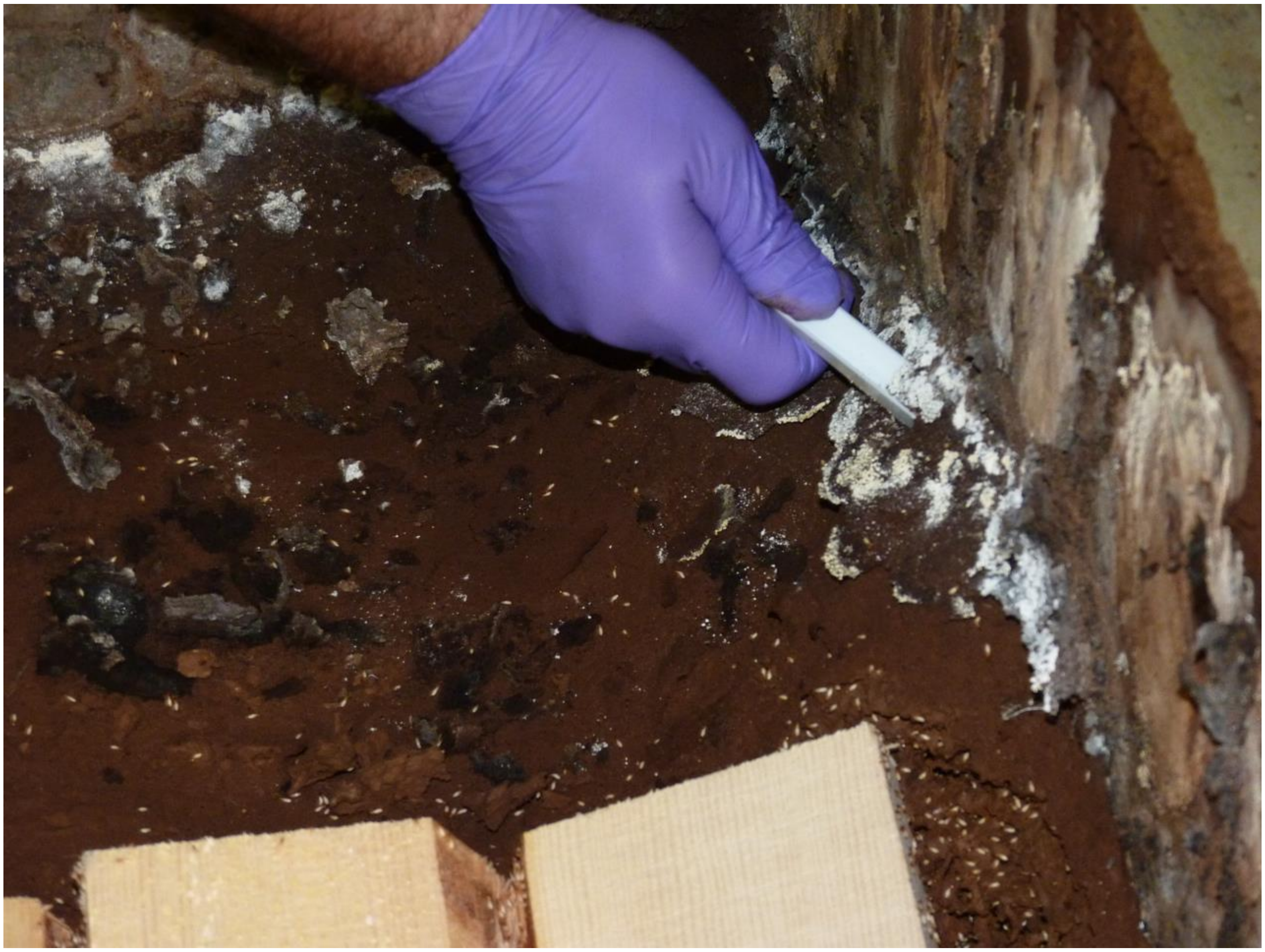

Supplementary Figure 7. Sampling of the nest material of a second *C. testaceus* colony facing a fungal infection at an early stage. The colony suffered to death and no corpse residues *e.g.* head capsules could be found in the nest at a late stage of the infection.

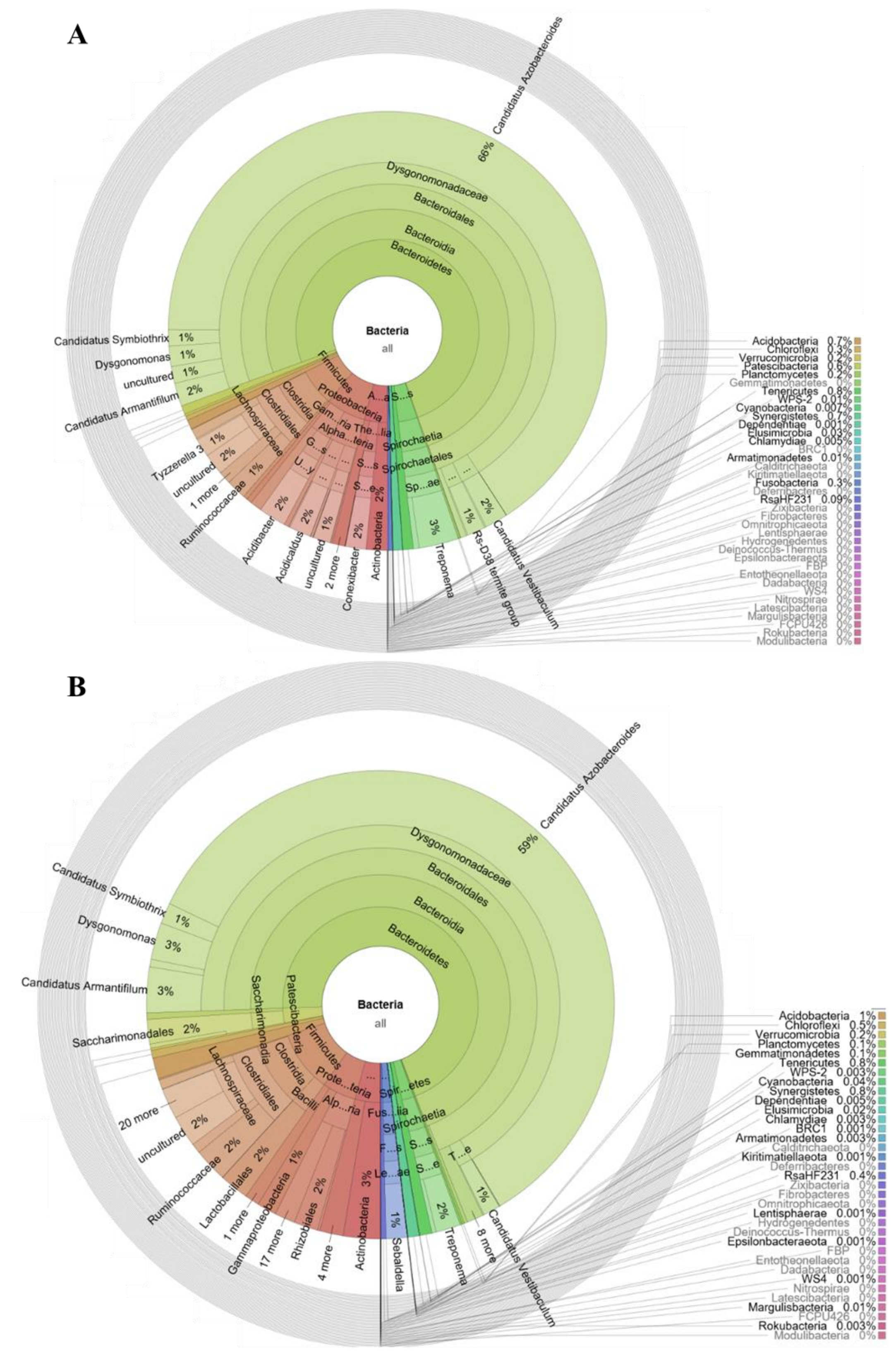

Supplementary Figure 8. Krona-chart depicting the microbiomes of *C. testaceus* termite soldiers from a healthy colony (**A**) and at an early stage of fungal infection (**B**). Unfortunately, no termites were found at the late stage of infection for the investigation of their bacterial microbiome.

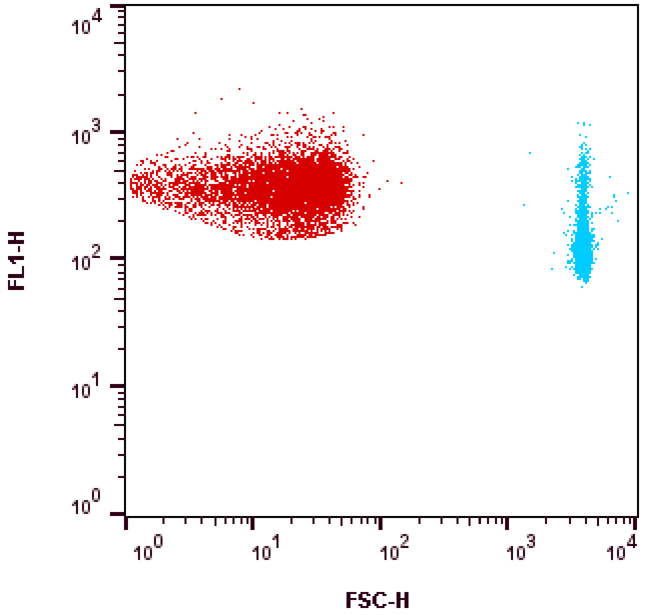

	Sample name	Count
(blue)	Beads	4506
(red)	*Coptotermes testaceus*1:1000	35814

Supplementary Figure 9. Dot plot used to calculate the amount of cells within the (Minisart®, 5 µm cellulose acetate syringe filter, Sartorius AG) the cell phase after nycodenz density gradient centrifugation. Cell count was done in triplicates, one representative Dot plot is shown. Cell staining using SYTO9 and FACS analysis was done according to manufacturer's protocol (Molecular Probes™, Bacteria Counting Kit, for flow cytometry, MP 0727).
